# Echoes of ease: Tracing the course of obsessive‐compulsive symptoms in the aftermath of a pandemic—Insights from a four‐year panel study

**DOI:** 10.1111/bjc.70015

**Published:** 2025-10-15

**Authors:** Lea Schuurmans, Anna Baumeister, Anja S. Göritz, Steffen Moritz, Franziska Miegel, Lena Jelinek

**Affiliations:** ^1^ Department of Psychiatry and Psychotherapy University Medical Center Hamburg‐Eppendorf Hamburg Germany; ^2^ Behavioral Health Technology University of Augsburg Augsburg Germany

**Keywords:** anxiety, C19, mental health, public health, SARS‐CoV‐2

## Abstract

**Objectives:**

The COVID‐19 pandemic affected mental health worldwide, including obsessive‐compulsive symptoms (OCS) and, specifically, contamination‐related OCS (C‐OCS). This study aimed to map these symptoms' trajectories over four years, thus providing the longest known observational timeline of OCS during and after a pandemic. Longitudinal data on OCS spanning this period offer the rare opportunity to examine how external crises intersect with symptom trajectories over time, offering insights into patterns of symptom fluctuation and remission.

**Design:**

We conducted a longitudinal panel study with 1,214 participants from the German general population assessed at the onset of the pandemic (T1), three months later (T2) and 12 months after T1 (T3), followed by annual assessments (T4, T5, T6).

**Methods:**

We measured OCS and C‐OCS using the Obsessive‐Compulsive Inventory‐Revised and calculated mixed‐effect models for repeated measurements.

**Results:**

Analyses indicated a significant but declining increase in OCS from T1 to T2, T3 and T4 (*d* = −.11 to −.05), with symptoms returning to baseline levels during annual assessments at T5 (*d* = −.02) and T6 (*d* = .03). By contrast, C‐OCS consistently and significantly decreased at each timepoint, indicating a steady reduction in symptom severity over the assessment period (*d* = .12 to .46).

**Conclusion:**

The initial OCS spike reflects stress and uncertainty in the population, while the steady C‐OCS improvement may be due to the normalization of hygiene practices over time. These patterns highlight the impact of pandemic‐related health management on C‐OCS. Factors affecting the general population that are linked to these symptoms can contribute to an individual's deterioration (for example promotion of hygiene practices). Understanding these dynamics contributes to a better understanding of C‐OCS and associated risk factors.

**Practitioner points**

- Extended observation: This study provides the longest assessment (4 years) of OCD symptoms during and after the COVID‐19 pandemic, offering valuable longitudinal data for clinical practice.
- General OCD symptoms: Findings indicate that general OCD symptoms spiked initially due to the pandemic but decreased over time, highlighting the importance of monitoring symptom fluctuations in response to external stressors.
- Contamination‐related symptoms: Contamination‐related OCD symptoms showed a faster recovery compared with other OCD symptoms, suggesting that specific symptom types may respond differently to pandemic‐related stress.

## INTRODUCTION

During the COVID‐19 pandemic, a large percentage of the world's population faced multiple challenges, including the risk of infection, social isolation, quarantines, lack of access to health services and economic insecurity (Broihanne et al., 2025; Faramarzi et al., 2024; Kleimeier et al., 2023; Panneer et al., 2022; Schäfer et al., 2022). Moreover, the pandemic's impact on mental health in both the general population and among people with preexisting mental illnesses has been a topic of public discussion since the first days of the pandemic (Rittmannsberger et al., 2023).

Research on past pandemics, such as the H1N1 or ‘swine flu’, the Zika virus and the Ebola outbreak, has shown consistent patterns in terms of their detrimental impact on mental health, particularly in relation to obsessive‐compulsive symptoms (OCS) and health anxiety (Dennis et al., 2021; Wheaton et al., 2012). In line with these past experiences, the COVID‐19 pandemic significantly affected mental health, especially in its first year but also in subsequent years. Fountoulakis et al. (2022) assessed an international sample (*N* = 55,589) from April 2020 to March 2021 and found increased levels of depression, anxiety and distress, as well as deteriorating family relations and daily life. Fourteen months into the pandemic, Rossi et al. (2023) and Schrempft et al. (2023) noted declining anxiety and depression, likely due to resilience and relaxed precautions.

Not only were affective and stress‐related symptoms impacted in the general population during the pandemic, but fear of contamination also became widespread and hygiene behaviours were encouraged by the World Health Organization. Recommended preventive measures, such as frequent handwashing and isolation, mirrored behaviours associated with obsessive‐compulsive disorder (OCD), particularly in the context of contamination, thus potentially intensifying existing symptoms (Jelinek et al., 2024; Zaccari et al., 2021).

Moreover, people without OCS were suddenly confronted with thoughts and behaviours that are routinely experienced and carried out by people with contamination‐related OCS (C‐OCS). This may potentially have led to an increase in the incidence of OCS in general and of C‐OCS in particular (Abba‐Aji et al., 2020). Characteristics of these symptoms include unwanted, intrusive thoughts and fears (obsessions), which lead to repetitive, ritualized behaviours (compulsions). Various studies have demonstrated the impact of the COVID‐19 pandemic on individuals with OCS of any kind, revealing an initial worsening of symptoms during the pandemic at the beginning (Abba‐Aji et al., 2020; Guzick et al., 2021; Jelinek et al., 2024; Linde et al., 2022; Tanır et al., 2020). However, Tandt et al. (2021) reported an initial increase in OCS coinciding with the pandemic's onset, followed by a decrease over the course of 2020.

The long‐term trajectory of OCS post‐pandemic is worth examining as long‐lasting consequences of global health crises are anticipated, and OCD is known to be associated not only with impaired quality of life but also with an elevated risk of suicide (Fernández de la Cruz et al., 2017; Hollander et al., 2010). Apart from the psychological distress caused, for example, by excessive cleaning rituals, physical impairments such as dermatitis and eczema have been noted (Mavrogiorgou et al., 2015). The existing literature examines the evolution of OCS up to 2021 or 2022. Reviews show an increase in specific symptoms such as washing, cleaning and other OCS affecting both clinical cohorts and the general population, suggesting the persistence of the previously mentioned short‐term effects (Pozza et al., 2024; Pugi et al., 2023). Some studies included in these reviews, like the longitudinal study by Grøtte et al. (2022), reported a rise in C‐OCS at the beginning of the pandemic in the general population, with symptoms slightly decreasing nine months in but not going back down to pre‐pandemic levels; additionally, Wheaton, Ward, et al. (2021) showed that the majority of individuals with self‐identified OCD reported worsening symptoms during the pandemic. Conversely, some longitudinal studies indicate a decrease in OCS severity for some individuals with OCD or subclinical OCS as the pandemic unfolded (Schwartz‐Lifshitz et al., 2021; Tandt et al., 2021; Zaccari et al., 2021), suggesting a trend towards symptom improvement and recovery over time. Overall, findings on the course of OCS are mixed (Grant et al., 2022), and the after‐effects of the pandemic in the current post‐pandemic period have not yet been thoroughly investigated, a notable gap. To our knowledge, this paper is the first attempt to assess the long‐term effects of the pandemic over its entire course and to critically evaluate the predominant expectation of symptom exacerbation.

As we navigate the post‐COVID‐19 pandemic period, understanding the trajectory of OCS and potential signs of recovery is crucial to identify specific periods when targeted support might be necessary and reduce the duration of potential suffering due to OCS. This article aims to explore the longitudinal changes in OCS during and after the COVID‐19 pandemic, shedding light on the initial exacerbation and subsequent recovery observed in a population‐based sample.

## METHODS

### Recruitment and procedure

As previously described, we invited *N* = 14,285 members of WisoPanel® (www.wisopanel.net) to participate in a web‐based assessment (the Obsessive‐Compulsive Inventory‐Revised [OCI‐R]) between 21 March and 30 March 2020, during the first COVID‐19 lockdown in Germany (Jelinek et al., 2021). WisoPanel has members from all walks of life and is described in detail elsewhere (Göritz, 2012; Göritz et al., 2021). Of the invitees, *n* = 2727 participated in the study. The responses of *n* = 2255 participants are included in previous analyses of the data set. Participants who did not complete the OCI‐R washing subscale, showed stereotypical response patterns or self‐reported responding untruthfully were excluded (i.e. people indicating in the final question that they had answered the questions untruthfully; see Jelinek et al., 2021). We invited the remaining participants to a second assessment three months later between 22 June and 30 June 2020 (T2) (Jelinek et al., 2021, 2022), soon after the easing of the first lockdown restrictions in Germany. A third assessment was conducted 12 months after T1, between 3 April and 12 April 2021 (T3) (Jelinek et al., 2024), 24 months after T1 (T4), 36 months after T1 (T5) and 48 months after T1 (T6). The online platform Unipark/Questback® (Globalpark AG) was used for all assessments. To maintain participant engagement across the six annual waves, participants received incentives at each timepoint. These included self‐help resources (a self‐esteem‐boosting manual at T1, cognitive‐behavioural therapy strategies for pandemic mental health at T2, a sleep improvement manual at T5 and evidence‐based techniques for coping with negative thoughts at T6) available via download and points within the WiSoPanel's loyalty program (10 points each at T3 and T4, worth 1€). All materials were designed to support participants' well‐being during the study period.

During the third assessment (2021, T3), Germany was no longer locked down, but many restrictions were still in place. These included the ‘federal emergency brake’ that would be activated if the 7‐day incidence was higher than 100 in any region (which would lead, for example to a curfew and restrictions on store openings). At the beginning of the fourth assessment (T4), the restrictions were gradually relaxed and participation in social life was once again possible for people who had been vaccinated against, recovered from or tested negative for COVID‐19. At this point, many protective measures were only applied in high‐incidence regions. At the time of the fifth assessment (T5), the COVID‐19 protection restrictions in Germany were entirely lifted and were not reactivated until the sixth assessment (T6). An overview of the timeline of COVID‐19 restrictions in Germany is given in Figure S2.

**FIGURE 1:**
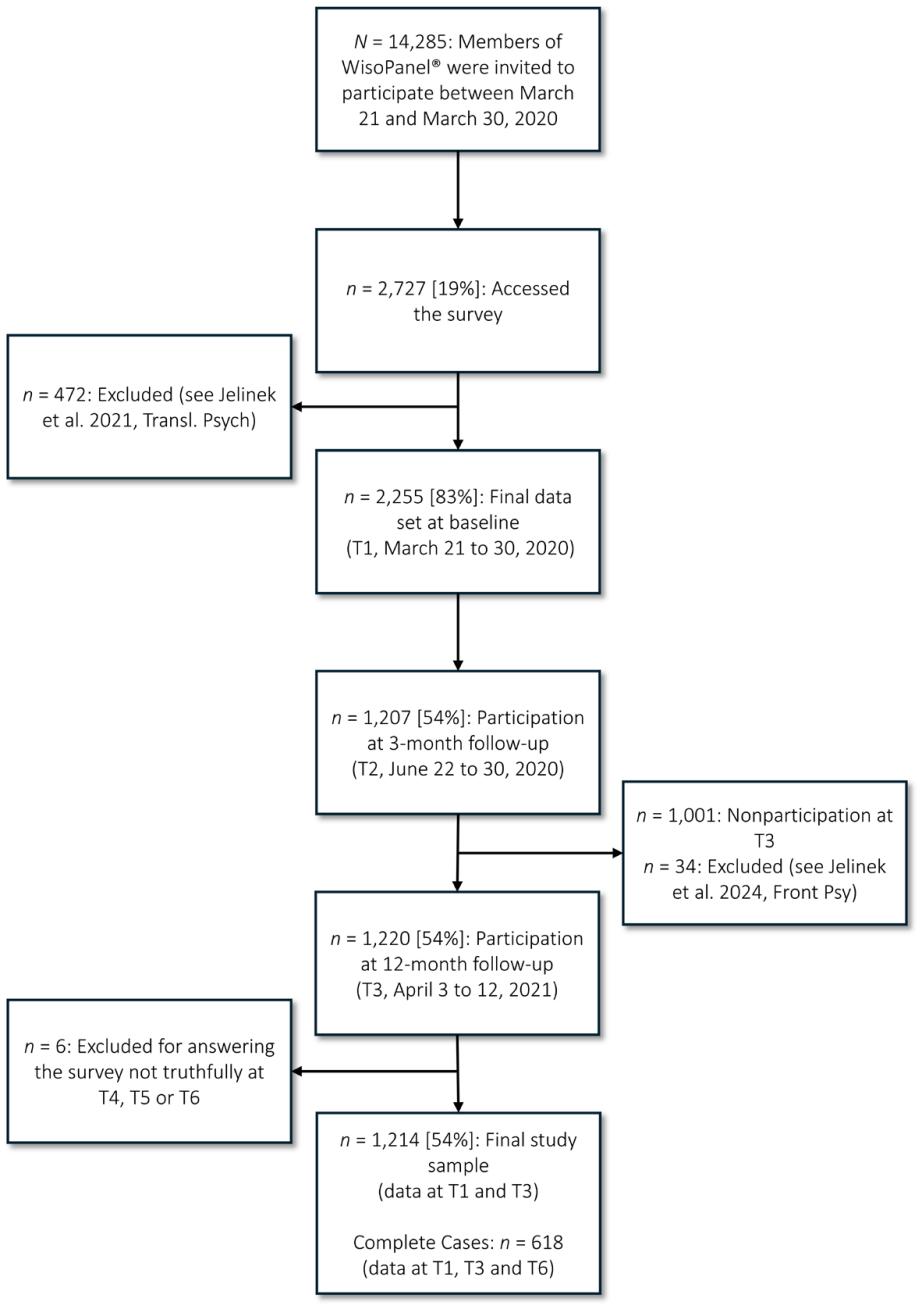
Flow chart of the participants through the study.

In the current study, we included participants with available data at T1 and T3 and excluded participants who reported having answered untruthfully, leading to a final sample of *N* = 1214 participants. For sample sizes at the different assessments, see Figure 1, and for a visualization of the missing data, see Figure S1.

### Assessment

#### Obsessive‐Compulsive Inventory‐Revised (OCI‐R)

OCS were measured with the OCI‐R (Foa et al., 2002; Gönner et al., 2008). Six subscales—washing, obsessing, checking, neutralizing, hoarding and ordering—were used to evaluate different domains of symptoms. The OCI‐R is sensitive enough to detect change (Abramowitz et al., 2005) and has shown good psychometric properties (Gönner et al., 2007). The washing subscale (Items 5, 11 and 17) assesses difficulty in touching objects and excessive washing and therefore captures C‐OCS. In the study at hand, the internal consistency was determined for the OCI‐R; Cronbach's α ranged between .92 and .93 for all six assessments. The internal consistency of the washing subscale ranged between .72 and .83. Recommended cut‐off scores for the German version were 18 for the total score and 3 for the washing subscale (Gönner et al., 2009).

### Data analysis

A mixed model for repeated measurements (MMRM) was employed to evaluate the impact of time on symptom changes from T1 (OCI‐R total score, washing subscale), with timepoints nested within individuals and using an unstructured structure. This methodological approach is increasingly favoured in analyses of longitudinal data due to its ability to effectively model correlation between repeated measures on the same subject and its flexibility in capturing time effects (Andersen & Millen, 2012; Gueorguieva & Krystal, 2004). The covariance structure of the MMRM is particularly useful for handling missing data appropriately and providing accurate estimates in longitudinal studies (Gosho et al., 2015; Mallinckrodt et al., 2004). Covariates included in the model were age, gender, symptom severity at T1 and employment. Analyses were conducted using R (R Core Team, 2024), especially the *mmrm* package (Bove et al., 2024). Marginal means were derived from the model at each timepoint, allowing for a detailed examination of the temporal trends in symptom changes. The comparison of marginal means between separate consecutive timepoints allows for a comprehensive analysis of the temporal pattern in symptom changes. To evaluate the significance of these differences, *p*‐values for the marginal means comparisons were adjusted using the Holm–Bonferroni method for multiple comparisons (Holm, 1979). To evaluate the size of the time effect, Cohen's *d* (with *d* ≈ .2, *d* ≈ .5 and *d* ≈ .8, corresponding to small, medium and large effects) was calculated.

## RESULTS

### Sample

Demographics were as follows: mean age of 55.49 years (*SD* = 13.71), *n* = 666 women (54.8%) and *n* = 712 (58.0%) with a university entrance qualification. Furthermore, *n* = 812 (61.2%) stated they were currently employed, while the remaining participants were unemployed, retired, students or on parental leave.

### Longitudinal course of OCS

**FIGURE 2:**
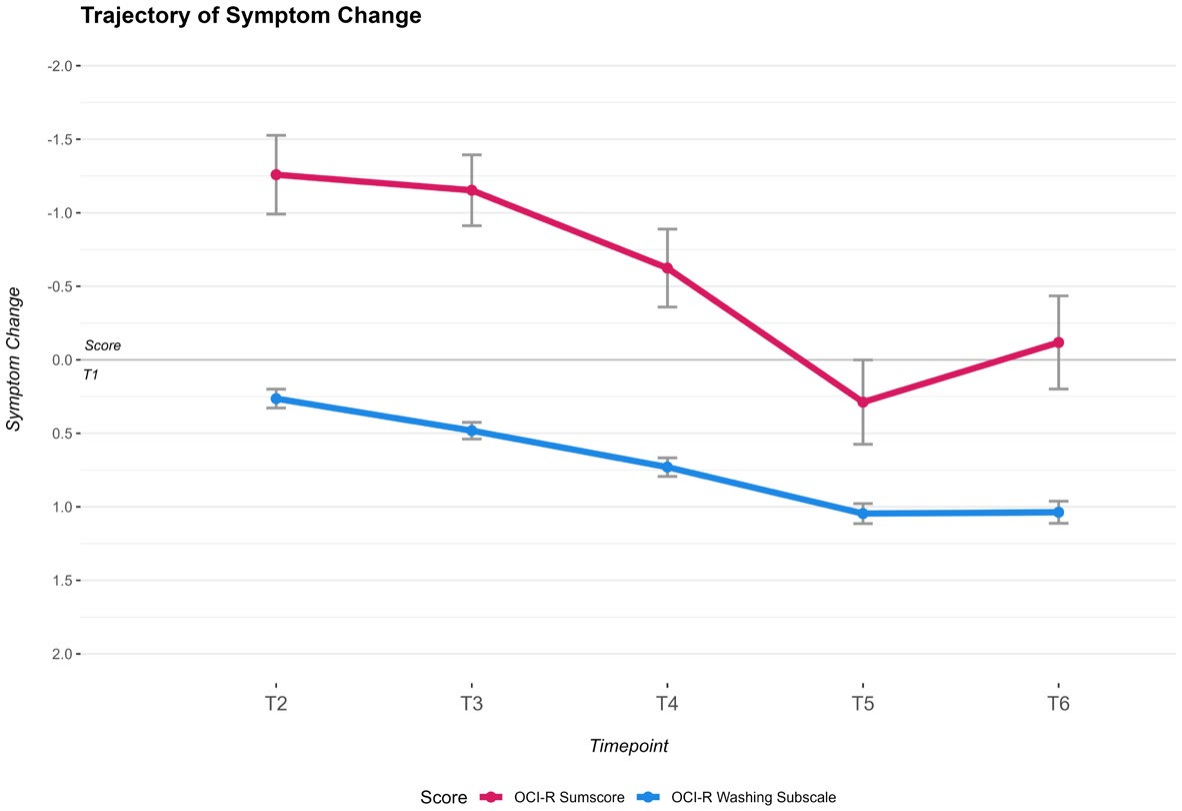
Change from T1 in OCS and C‐OCS.OCI‐R sumscore and OCI‐R washing subscale means with standard error, based on MMRM of the sample 3 months (T2), 12 months (T3), 24 months (T4), 36 months (T5) and 48 months (T6) after T1.

The descriptive evaluation of the sum and change scores revealed an increase in OCS symptoms from T1 to T3, followed by a subsequent decrease, with T6 scores falling below the T1 level (Table 1). The evaluation of change scores compared with T1 showed fluctuation in symptom change over time (Table 2). The least square means analysis for each timepoint showed a significant increase of OCS at T2, T3 and T4 (*p* < .05), at a size ranging from *d* = −.11 to −.05, indicating fluctuations in symptom severity over time. However, the least square means for T5 and T6 were not significant, suggesting the stabilization of symptom severity at these later timepoints (Table 2). To account for potential confounding, age, gender, education, employment status and T1 symptom severity were included as covariates in all models. Only symptom severity at T1 emerged as a significant predictor of subsequent symptom change in OCS across timepoints (*p* < .001), while none of the other covariates were significant. The fixed effects table of the model can be found in the appendix (Table S1). Further insights were gained through the contrasts between consecutive timepoints. There was a significant decline in OCS between T4 and T5 (*p* < .05), highlighting specific transitions in symptom severity during the study period. By contrast, the differences between T2 and T3, T3 and T4, and T5 and T6 were not significant (*p* > .07), indicating periods of relative stability in symptom severity between these timepoints (Table 2). A visualization of the trajectory of change in OCS and C‐OCS is given in Figure 2.

**TABLE 1:** Sample size and OCS measured by the OCI‐R over time.

Timepoint	*N* (%)	OCI‐R sumscore *M* (*SD*)	OCI‐R washing subscale *M* (*SD*)
T1	1,204 (100%)	11.96 (11.03)	2.32 (2.54)
T2	882 (73%)	12.93 (11.50)	1.99 (2.38)
T3	1,204 (100%)	13.02 (11.25)	1.81 (2.31)
T4	885 (73%)	12.36 (11.54)	1.56 (2.20)
T5	747 (62%)	11.36 (10.71)	1.21 (1.99)
T6	620 (51%)	11.63 (11.17)	1.23 (2.08)

*Note*: Only participants with available data at T1 and T3 were included.

Abbreviations: OCI‐R, Obsessive‐Compulsive Inventory‐Revised; T1, first assessment; T2, 3 months after the T1 assessment (T1); T3, 12 months after T1; T4, 24 months after T1; T5, 36 months after T1; T6, 48 months after T1.

**TABLE 2:** EMM for change in OCS and C‐OCS over time from MMRM with pairwise consecutive comparison of the EMM.

Outcome	Timepoint	EMM (*SE*)	95% CI		*t*	*p*	*d*	Contrast (*SE*)	95% CI		*t* ratio	*p*
			LL	UL								
OCI‐R	T2	−1.201 (.250)	−1.692	−.711	−4.803	<.001	−.11					
	T3	−1.138 (.241)	−1.610	−.665	−4.722	<.001	−.10	.063 (.240)	−.537	.664	.264	.792
	T4	−.612 (.270)	−1.142	−.083	−2.269	.023	−.05	.525 (.238)	−.070	1.121	2.207	.083
	T5	.253 (.287)	−.310	.817	.883	.378	.02	.866 (.267)	.198	1.534	3.246	.005
	T6	−.394 (.314)	−.856	.373	−1.004	.206	−.04	−.647 (.293)	−1.381	.087	−2.209	.083
OCI‐R	T2	.278 (.065)	.151	.405	4.295	<.001	.12					
Washing	T3	.489 (.059)	.608	.859	11.466	<.001	.33	.211 (.061)	.059	.363	3.479	.001
Subscale	T4	.733 (.064)	.608	.859	11.466	<.001	.33	.244 (.060)	.095	.393	4.094	<.001
	T5	1.034 (.063)	.910	1.157	16.435	<.001	.46	.301 (.059)	.152	.450	5.053	<.001
	T6	.394 (.069)	.839	1.110	14.100	<.001	.43	−.060 (.062)	−.215	.096	−.957	.339

*Note*: Adjusted *p*‐values were calculated using the Holm–Bonferroni method to control for multiple comparisons.

Abbreviations: CI, confidence interval; *d*, Cohen's *d*; EMM, estimated marginal means; LL, lower limit; MMRM, mixed models for repeated measurements; OCI‐R, Obsessive‐Compulsive Inventory‐Revised; T2, 3 months after the first assessment (T1); T3, 12 months after T1; T4, 24 months after T1; T5, 36 months after T1; T6, 48 months after T1; UL, upper limit.

### Longitudinal course of C‐OCS

The analysis of change scores in the washing subscale of the OCI‐R, reflecting the alteration in C‐OCS compared with T1 at the start of the COVID‐19 pandemic, revealed a consistent pattern. The descriptive evaluation of the sum demonstrated a decrease in C‐OCS from T1 to T6, when all scores fell below the T1 level (Table 1). The least square means for each timepoint's change score showed that all timepoints from T2 to T6 were statistically significant (*p* < .01), with an increasing effect from *d* = .12 to *d* = .46. This indicates a consistent impact on C‐OCS across these timepoints, reflecting a significant reduction in symptom severity compared with T1 (Table 2). The fixed effects table of this model can also be found in the appendix; none of the covariates, except for symptom severity at T1 (*p* < .001), were significant predictors of changes in C‐OCS across timepoints (Table S1). Further analyses through contrasts between consecutive timepoints yielded significant differences (*p* < .05) between all consecutive timepoints except from T5 to T6 (*p* = .68) (Table 2). Specifically, there was a significant decline in C‐OCS between T2 and T3, and T3 and T4, as well as between T4 and T5, highlighting shifts in C‐OCS during these intervals. However, the lack of significance between T5 and T6 suggests a stabilization or plateauing of symptom severity at these later timepoints (Table 2).

## DISCUSSION

This longitudinal study represents the longest period of observation of OCS to date, extending from during to after the COVID‐19 pandemic, and it provides insights into mental health responses, specifically OCS trajectories, to a global health crisis. The initial exacerbation of symptoms observed during the pandemic aligns with findings from other studies with a shorter observation period that demonstrated a worsening of OCS, particularly C‐OCS (Guzick et al., 2021; Tanır et al., 2020; Zaccari et al., 2021).

This study contributes to the literature on the impact of the COVID‐19 pandemic on OCS. While some studies report an exacerbation of symptoms and an increase in obsessive and compulsive behaviours during the pandemic (Alkhamees, 2021; Moreira‐de‐Oliveira et al., 2022), others highlight the adaptation of individuals with OCD to the challenges posed by the crisis (Blendermann et al., 2024; Sharma et al., 2024; Tulacı et al., 2022). The earlier results from this sample show that OCS increased slightly overall in the general population from March to June 2020 (Jelinek et al., 2021). While the prevalence of C‐OCS in March 2020 was significantly higher than pre‐pandemic levels in 2014, the symptom burden of washing behaviour decreased steadily through June 2020 and April 2021 (Jelinek et al., 2024). This pattern of an initial symptom surge followed by gradual normalization mirrors the trajectory observed in previous pandemics, including SARS, Ebola, Zika and H1N1, which were associated with increased anxiety, contamination fears and obsessive‐compulsive behaviours, particularly in individuals with predisposing cognitive vulnerabilities (Dennis et al., 2021; Wheaton et al., 2012). Recent findings from the COVID‐19 pandemic emphasize the role of intolerance to uncertainty and contagion of emotions through media exposure in the amplification and maintenance of OC‐related distress (Dennis et al., 2021; Wheaton, Ward, et al., 2021; Wheaton, Messner, & Marks, 2021). These findings support the idea that symptom fluctuations during pandemics are characterized by both situational stressors and cognitive‐affective vulnerabilities. Consistent with this, the previous work suggests that transdiagnostic mechanisms such as unrealistic pessimism about severe illness and experiential avoidance contribute to the maintenance of C‐OCS in the general population (Jelinek et al., 2021, 2022).

The study's results based on the sample's data up to April 2024 indicate that there are differences in the intensity and persistence of the impact the pandemic had on OCS in general and C‐OCS specifically over the course of four years. Notably, the C‐OCS demonstrate a faster recovery compared with OCS in general, indicating distinct pathways of recovery for different symptom domains (Pugi et al., 2023). The faster recovery of C‐OCS could be attributed to the notion that COVID‐19 was found to be transmitted primarily through aerosols (Noorimotlagh et al., 2021), which cannot be effectively prevented by handwashing alone but is reduced by social distancing and isolation. This understanding may have led individuals to re‐evaluate the efficacy of excessive handwashing, contributing to a quicker decrease in C‐OCS related to hand hygiene. Conversely, isolation strategies and uncertainties during the pandemic could have perpetuated the increase in general OCS (Blendermann et al., 2024; Cardoș, 2024; D'Urso, 2024).

This is buttressed by the consideration that different domains of OCD are associated with specific avoidance behaviour in regard to social interaction (Starcevic et al., 2011). Due to isolation and social distancing, individuals may have experienced fewer triggers for C‐OCD symptoms, so associated behaviour did not manifest; the widely reported increase in these symptoms could therefore also be considered an artefact of the recommended enhanced hygiene behaviours. Nevertheless, ongoing stress, fear and unpredictability induced by the pandemic may have fuelled obsessions and compulsions beyond contamination‐related concerns, leading to a slower recovery trajectory overall OCS (Akın‐Sarı et al., 2022; Linde et al., 2022).

The difference in the recovery patterns shed light on the complex relationship between environmental factors, public health messaging and individual coping mechanisms during a global crisis. Rapid adaptation to new information about disease transmission and the adjustment of hygiene practices may have supported a swifter decrease in C‐OCS, highlighting the importance of context‐specific interventions and information tailored to the changing understanding of health threats (Juliana, 2024; Lattie & Stamatis, 2022; Tulacı et al., 2022). However, the data from this panel study reveal a trajectory of symptom recovery two years post‐pandemic, suggesting resilience and adaptive responses among individuals with OCS, which aligns with previous findings on depression and anxiety (Rossi et al., 2023; Schrempft et al., 2023). In addition to the main symptom trajectories, our models also took into account several covariates, including age, gender, educational level, employment status and symptom severity at the start of the study. Of these factors, only symptom severity at the start of the study was associated with subsequent change in (C‐)OCS, while the other covariates had no significant effect. This suggests that the overall trajectories were robust across all demographic subgroups when these potential influences were controlled for. Moving forward, it is essential to consider the implications of these findings for public mental health support. While society and health systems have gained insights from the COVID‐19 pandemic, the risk of future pandemics (Fauci & Folkers, 2023) emphasizes the need to prepare not only for disease management but also for the profound impacts on mental health. A deeper understanding of the trajectory of OCS is crucial due to the considerable impairment these symptoms may cause. By adapting public health management to the observed trajectories of OCS, the duration of suffering caused by OCS could potentially be reduced. For example, prevention programs proposed by Cardoș (2024), who suggested psychoeducation and resilience development, need to be investigated, expanded and made routine. By using insights from longitudinal studies and systematic reviews, mental health professionals can enhance their approaches to managing OCS in the aftermath of global health crises, thus fostering resilience and well‐being in the world's population.

### Limitations

A major limitation of our study is the attrition of participants over time; almost 50% of the original sample was lost at T6 (48 Months after T1). Even though high attrition is a known problem in long‐term, voluntary longitudinal studies, it may introduce bias and limit the generalizability of our results. Nevertheless, with the substantial sample size of 1214 participants, the MMRM addressed this issue, ensuring robust and reliable analysis of the dataset. Although we assume here that missing data are missing at random (MAR) and have accounted for this analytically, we cannot rule out the possibility that dropouts are related to unmeasured factors that influenced symptom progression. Second, assessments were conducted remotely rather than in person. This was the only approach possible due to pandemic‐related restrictions. To mitigate potential issues, cookies were used to prevent duplicate responses, and individuals with systematic response patterns were excluded. Third, another limitation is that we relied on a single self‐report measure (OCI‐R), which can be subject to bias. However, we chose to use the OCI‐R because it is a widely used and well‐validated instrument in OCD research, allowing comparison with existing studies. In addition, limiting the number of measurements helped to reduce participant burden, which was an important consideration given the panel design of our study. Finally, this study focused on self‐reported OCS rather than a clinical diagnosis. Establishing clearer insights into the development of psychopathology would necessitate clinical evaluations by mental health professionals. Assessing the general population, rather than focusing on individuals with clinical OCD, provides a broader understanding of how OCS manifest and fluctuate in a non‐clinical context against the background of a global health crisis, making the insights more applicable to the general population. Nevertheless, future research could expand on these findings by investigating whether individuals with an existing OCD diagnosis experience a different symptom trajectory compared with the general population in the context of this pandemic or comparable future scenarios.

## CONCLUSIONS

The differential recovery rates shown in C‐OCS and general OCS post‐pandemic show the pathways to symptom decrease. By highlighting the adaptive responses and resilience demonstrated by individuals in the face of adversity, this research contributes to a deeper understanding of mental health dynamics in the aftermath of a global health crisis. While the overarching message of recovery and resilience demonstrates the human capacity to overcome challenges and emerge stronger on the journey towards mental well‐being, it is important to recognize that any amount of time spent experiencing OCS represents a marked impairment of quality of life. Obsessive thoughts and compulsive actions create profound distress as individuals endure persistent, anxiety‐inducing ideas that feel overwhelmingly real, coupled with the urge to perform time‐consuming rituals to ease these feelings (APA, 2013). Therefore, it remains essential to search for possible ways to prevent OCS and to hasten individuals' recovery from OCS through appropriate support.

## AUTHOR CONTRIBUTIONS

**Lea Schuurmans:** Conceptualization; methodology; formal analysis; data curation; writing – original draft; writing – review and editing; visualization. **Anna Baumeister:** Methodology; writing – review and editing. **Anja S. Göritz:** Resources; data curation; writing – review and editing. **Steffen Moritz:** Writing – review and editing. **Franziska Miegel:** Writing – review and editing. **Lena Jelinek:** Conceptualization; data curation; supervision; project administration; writing – review and editing.

## CONFLICT OF INTEREST STATEMENT

The authors declare that they have no known competing financial interests or personal relationships that could have appeared to influence the work reported in this paper.

## ETHICS STATEMENT

The authors assert that all procedures contributing to this work complied with the ethical standards of the relevant national and institutional committees on human experimentation and with the Helsinki Declaration of 1975, as revised in 2008. Approval was granted by the Local Psychological Ethics Committee (LPEK; #LPEK‐0129, #LPEK‐0298). Informed consent was obtained from all subjects and/or their legal guardian(s).

## TRANSPARENCY

For previous publications on the OCS data of this sample at T1 and T2, see Jelinek et al. (2021, 2022, 2024).

## Supporting information


Appendix S1.

## Data Availability

The data that support the findings of this study are available on request from the corresponding author. The data are not publicly available due to privacy restrictions (German GDPR).
