# Supplementary material for: Echoes of ease: Tracing the course of obsessive‐compulsive symptoms in the aftermath of a pandemic—Insights from a four‐year panel study
Source: Br J Clin Psychol. 2025 Oct 15;65(1):236–49. doi: 10.1111/bjc.70015 (PMC12889211; doi:10.1111/bjc.70015)
Supplement: Supplementary file 1 — Appendix S1. [file BJC-65-236-s001.docx]

**Supplement Material**

**
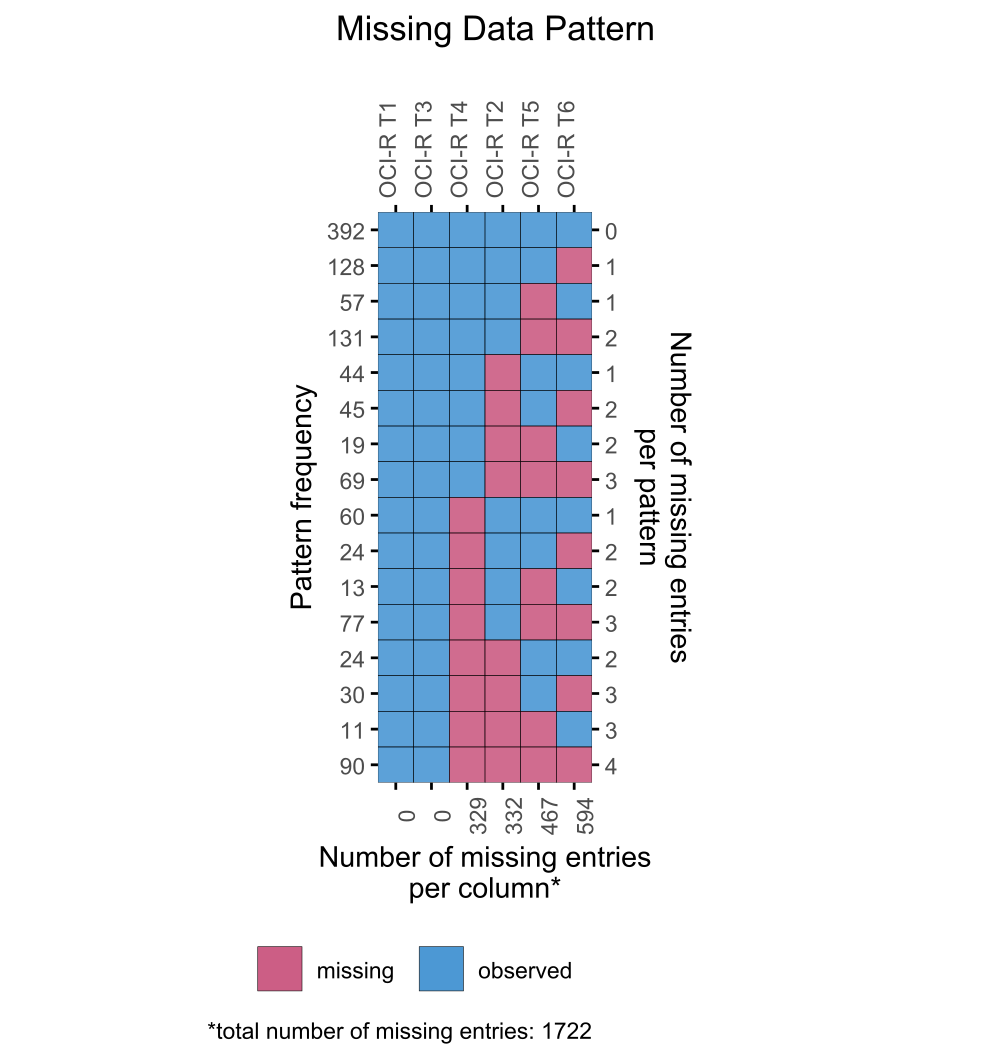
**

**Fig. S1.** Missing data pattern. OCI-R = Obsessive-Compulsive Inventory-Revised (OCI-R). Scores over the course of the study. T1 = first assessment, T2 = 3 months after T1, T3 = 12 months after T1, T4 = 24 months after T1, T5 = 36 months after T1, T6 = 48 months after T1.


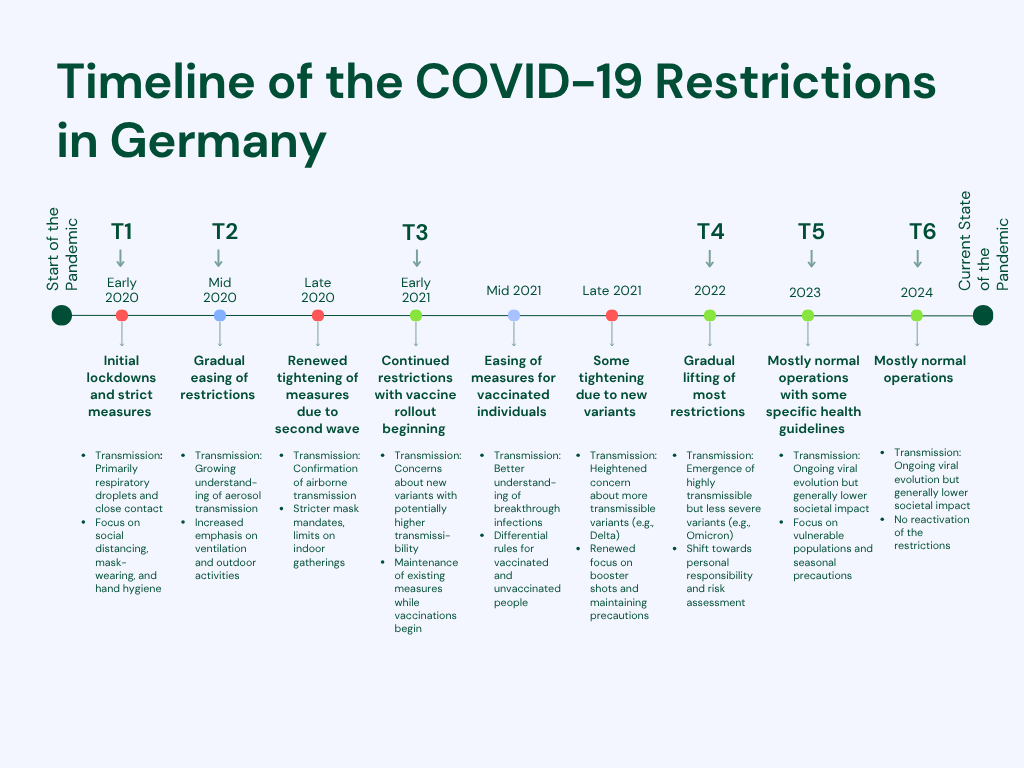


**Fig. S2**. Timeline of the COVID-19 Restrictions in Germany from 2020 to 2024. Transmission refers to the understanding of transmission at the time in question.

|  | **OCI–R Sumscore** | | | | | | | | | | **OCI–R Washing Subscale** | | | | | | |
| --- | --- | --- | --- | --- | --- | --- | --- | --- | --- | --- | --- | --- | --- | --- | --- | --- | --- |
| Predictors | | Estimates  *(SE)* | | *CI* | | *t* | | *p* | | | Estimates  *(SE)* | *CI* | | | *t* | | *p* |
|  |  | | *LL* | | *UL* | |  | |  |  | | *LL* | *UL* |  | |  | |
| (Intercept) | –1.174  (0.167) | | 0.098 | | 0.752 | | 2.547 | | 0.011 | 0.425  (0.458= | | –2.073 | –0.274 | –2.560 | | 0.011 | |
| Sex | 0.203  (0.093) | | –0.332 | | 0.033 | | –1.610 | | 0.108 | –0.149  (0.390) | | –0.563 | 0.968 | 0.519 | | 0.604 | |
| Education | –0.288  (0.093) | | –0.104 | | 0.262 | | 0.842 | | 0.400 | 0.079  (0.391) | | –1.055 | 0.479 | –0.737 | | 0.461 | |
| Age | –0.142  (0.054) | | –0.076 | | 0.137 | | 0.560 | | 0.576 | 0.030  (0.229) | | –0.591 | 0.306 | –0.623 | | 0.533 | |
| Employment | 0.030  (0.111) | | –0.143 | | 0.294 | | 0.680 | | 0.496 | 0.076  (0.466) | | –0.883 | 0.944 | 0.065 | | 0.948 | |
| OCI–R T1 | 2.909  (0.045) | | 1.285 | | 1.462 | | 30.394 | | <0.001 | 1.373  (0.190) | | 2.536 | 3.282 | 15.295 | | <0.001 | |
| Time (3) | 0.063  (0.061) | | 0.092 | | 0.330 | | 3.479 | | 0.001 | 0.211  (0.240) | | –0.408 | 0.534 | 0.264 | | 0.792 | |
| Time (4) | 0.589  (0.068) | | 0.322 | | 0.588 | | 6.715 | | <0.001 | 0.455  (0.265) | | 0.069 | 1.108 | 2.224 | | 0.026 | |
| Time (5) | 1.455  (0.068) | | 0.622 | | 0.889 | | 11.087 | | <0.001 | 0.756  (0.285) | | 0.895 | 2.014 | 5.102 | | <0.001 | |
| Time (6) | 0.807  (0.070) | | 0.558 | | 0.834 | | 9.922 | | <0.001 | 0.696  (0.286) | | 0.247 | 1.368 | 2.827 | | 0.005 | |

**Tab. S1** Fixed Effects for Change in OCS and C-OCS over Time from MMRM

*Note. OCI-R = Obsessive-Compulsive Inventory-Revised; T1 = first assessment, Time 3 = 12 months after T1, Time 4 = 24 months after T1, Time 5 = 36 months after T1, Time 6 = 48 months after T1; MMRM = mixed models for repeated measurements; CI = confidence interval, LL = lower limit, UL = upper limit.*
